# Effect of Smartphone Use on Intraocular Pressure

**DOI:** 10.1038/s41598-019-55406-1

**Published:** 2019-12-11

**Authors:** Eun Ji Lee, Hyunjoong Kim

**Affiliations:** 1 Department of Ophthalmology, Seoul National University Bundang Hospital, Seongnam, Korea; 2 Department of Applied Statistics, Yonsei University, Seoul, Korea

**Keywords:** Glaucoma, Optic nerve diseases

## Abstract

The rapidly increasing utilization of smartphones makes ophthalmic problems associated with their use an important issue. This prospective study aimed to determine whether using a smartphone to view visual material is associated with a change in the intraocular pressure (IOP), and to determine which groups of factors best predict the time-dependent increase in IOP with smartphone use. This study included 158 eyes (127 glaucomatous and 31 healthy eyes) recruited from Seoul National University Bundang Hospital. Participants performed a sustained fixation task consisting of watching a movie on a smartphone screen for 30 minutes continuously at a viewing distance of 30 cm. A small but statistically significant time-dependent increase in IOP was observed while viewing a movie on a smartphone, being 10.6 ± 3.1, 11.0 ± 3.3, 11.2 ± 3.4, and 11.6 ± 3.5 mmHg before and 5, 10, and 30 minutes after the fixation task, respectively (*P* < 0.0001). Recursive partitioning tree analysis revealed that a shallower anterior chamber (<2.32 mm) was the strongest predictive factor for faster time-dependent increase in IOP (0.68 mmHg/minute). A higher visual field mean deviation (≥–0.22 dB), and an older age (≥48 years) were the second and third most influential factors associated with the rate of IOP increase (0.59 and 0.15 mmHg/minute, respectively).

## Introduction

The use of smartphones has been increasing rapidly since their introduction in the late 2000s. It has been reported that approximately 70% of the on-line population in the United States was using smartphones in 2017^1,2^. The rate of smartphone penetration is similar in South Korea (71.5%), with smartphone use among the general population ranking the fourth highest worldwide^1^. It was recently estimated that >50% and 10% of smartphone users connect to the Internet for >30 minutes and >4 hours daily, respectively^3^. Moreover, the users of mobile devices reportedly spend >20 hours weekly using email, text messages, and social networking services, which indicates that they are heavily reliant on smartphones in their communication with other people^4^.

Smartphone overuse is linked to various ophthalmic problems, including eyestrain, ocular discomfort, dry eye, diplopia, and blurry vision^5–8^. A sensation of an increased intraocular pressure (IOP) is among the most frequent ophthalmic complaints after the prolonged use of a smartphone. However, the precise effect of smartphone use on IOP is unclear. It is likely that this reported symptom is related merely to tightness of the eyelid from dry eye. However, the possibility of an actual increase in IOP cannot be excluded since the close viewing of small text on a small screen (as during smartphone use) increases the accommodation and vergence demands^9^, which are potentially associated with an increase in IOP^10,11^. It is also possible that disturbance of the circadian rhythm by nighttime smartphone use interrupts the normal circadian changes in IOP^12–16^, which may eventually result in IOP increasing. Whichever the case is, the effect of smartphone use on IOP needs to be determined.

The purpose of the study was to determine how the effect of smartphone use affect IOP, which was achieved by applying a fixation task consisting of watching movie clips on a smartphone to both healthy subjects and glaucoma patients. The study then attempted to develop a prediction model for any time-dependent increase in IOP with smartphone use. To this end, recursive partitioning (rpart) tree analysis was applied^17^. The rpart is an interactive statistical tool that can separate a group into two subgroups repeatedly given some risk factors of interest. This approach makes it possible to create a prediction model based on the combination of factors that could best explain different rates of increase in IOP.

## Results

The study included 158 eyes (127 glaucomatous eyes and 31 healthy eyes) of subjects aged 51.4 ± 12.1 years. A hundred and six subjects were using farsighted glasses, and none were using nearsighted glasses. Eighteen subjects needed habitual refractive correction for near vision at the time of fixation task. The baseline clinical characteristics of the participants are given in Table 1.

**Table 1:** Baseline clinical characteristics.

Age, *years*	51.4 ± 12.1
Sex, *male*/*female*	98/60
Refractive error, *diopters*	−3.74 ± 4.51
Central corneal thickness, *µm*	548.6 ± 40.0
Anterior chamber depth, *mm*	2.93 ± 0.43
Axial length, *mm*	25.69 ± 1.89
Global RNFL thickness, *µm*	77.3 ± 15.1
Visual field MD, *dB*	−5.57 ± 6.02
Visual field PSD, *dB*	5.86 ± 4.56
Visual field index, %	86.6 ± 16.9
Glaucoma, *n*	127 (80.4%)
Diabetes mellitus, *n*	18 (11.4%)
Systemic hypertension, *n*	24 (15.2%)
Systolic blood pressure, *mmHg*	125.7 ± 15.3
Diastolic blood pressure, *mmHg*	77.3 ± 11.6

RNFL = retinal nerve fiber layer; MD = mean deviation; PSD = pattern standard deviation.

### Time-dependent changes in IOP during the smartphone fixation task

The linear mixed-effects model for repeated-measures data showed that the IOP gradually increased during the smartphone fixation task (Table 2). Significant elevation of IOP was observed at 5 minutes after starting the task, and kept increasing until the end of the 30-minute viewing period (*P* < 0.0001).

**Table 2:** Time dependent IOP change after reading and watching tasks.

Baseline	5 min	10 min	30 min	*P-value*
10.6 ± 3.1^a^	11.0 ± 3.3^b^	11.2 ± 3.4^b^	11.6 ± 3.5^c^	<0.0001

^a,b,c^Different superscripts indicate significant differences between the values.

### Factors associated with IOP increase during the smartphone fixation task

**Figure 1:**
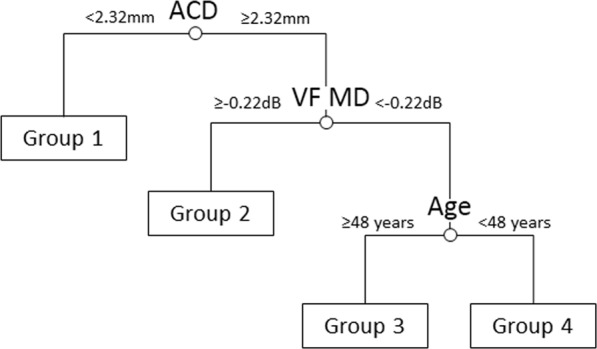
Recursive partitioning tree model stratifying the groups based on the factors best explaining the faster time-dependent increase in intraocular pressure. ACD, anterior chamber depth; VF MD, visual field mean deviation.

**Figure 2:**
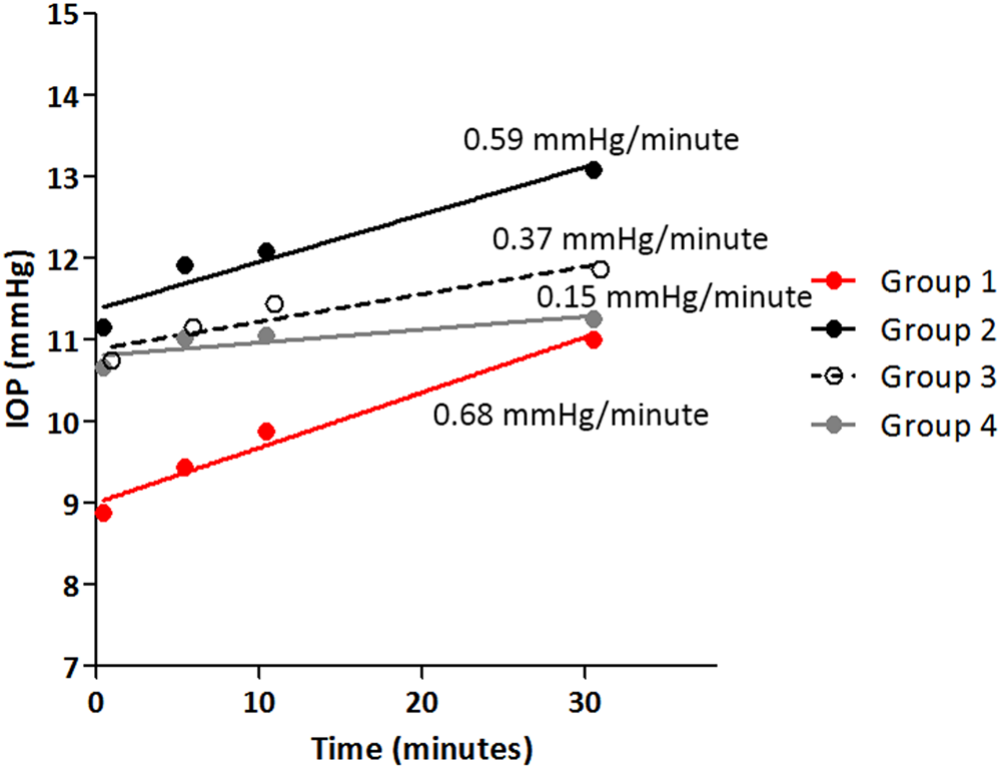
Time-dependent changes in intraocular pressure (IOP) in the groups as identified using the recursive partitioning tree model.

**Figure 3:**
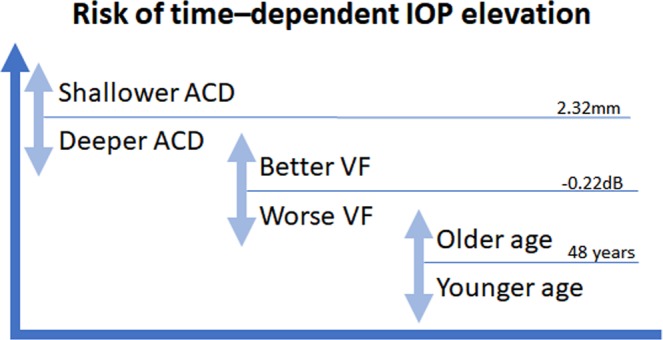
Schematic figure summarizing the main findings of the study.

Figure 1 shows a regression tree model determined based on the rpart tree analyses, which describes the factors that best predict the time-dependent increase in IOP while viewing the visual material. The analyses began with all respondents in the root node. Three nodes were then identified, resulting in four separate groups (Fig. 1). The first split was determined by ACD, with eyes with a smaller ACD (<2.32 mm) having the fastest time-dependent increase in IOP (0.68 mmHg/minute, Fig. 2). The second and third splits were determined by the VF mean deviation (MD) and age: subjects having a better VF MD (≥−0.22 dB) and those who were older (aged ≥48 years) had a faster time-dependent increase in IOP (0.59 and 0.37 mmHg/minute, respectively; Fig. 2). The time-dependent increase in IOP was statistically significant for all four groups (*P* ≤ 0.004, Table 3). Figure 3 summarizes the main findings of the study.

**Table 3:** Time dependent IOP change after smartphone task in each group divided based on the recursive partitioning tree model.

	Baseline	5 min	10 min	30 min	*P-value*
Group 1 (n = 16)	8.9 ± 2.3^a^	9.4 ± 2.3^b^	9.9 ± 2.3^c^	11.0 ± 3.1^d^	<**0.0001**
Group 2 (n = 13)	11.2 ± 3.4^a^	11.9 ± 3.4^b^	12.1 ± 3.8^b^	13.1 ± 4.5^c^	**0.001**
Group 3 (n = 68)	10.6 ± 3.0^a^	11.1 ± 3.5^b^	11.4 ± 3.6^c^	11.7 ± 3.4^d^	<**0.0001**
Group 4 (n = 61)	10.9 ± 3.3^a^	11.2 ± 3.3^b^	11.2 ± 3.4^b^	11.4 ± 3.6^c^	**0.004**

^a,b,c^Different superscripts in each group indicate significant differences between the values.

Statistically significant values are in bold face letters.

Comparisons between the groups classified in the rpart tree revealed that group 1 was more hyperopic, and had a shorter axial length and a smaller ACD; group 2 had a higher VF MD and lower PSD; group 4 was younger than the other three groups (Table 4).

**Table 4:** Comparison between the groups divided by recursive partitioning tree analysis.

	Group 1 (n = 16)	Group 2 (n = 13)	Group 3 (n = 64)	Group 4 (n = 58)	*P-value*	*Post-Hoc*
Slope of IOP change, *mmHg/min*	0.68 ± 0.58	0.59 ± 0.57	0.37 ± 0.58	0.15 ± 0.39	**0.001**	1 > 2 > 3 > 4
Age, *years*	57.9 ± 10.7	53.9 ± 9.0	59.6 ± 7.6	39.6 ± 6.7	<**0.001**	1,2,3 > 4
Sex, *male*/*female*	9/7	12/1	39/25	36/22	0.154	
Baseline IOP, *mmHg*	8.9 ± 2.3	11.2 ± 3.4	10.7 ± 3.1	10.9 ± 3.3	0.134	
Refractive error, *diopters*	0.69 ± 1.11	−3.38 ± 2.05	−4.36 ± 5.96	−4.51 ± 2.73	<**0.001**	1 > 3,4
Central corneal thickness, *µm*	559.4 ± 45.2	554.9 ± 54.7	555.1 ± 27.8	537.6 ± 43.7	0.053	
Anterior chamber depth, *mm*	2.18 ± 0.11	3.01 ± 0.48	2.93 ± 0.36	3.12 ± 0.33	<**0.001**	1 < 2, 3, 4, 3 < 4
Axial length, *mm*	23.57 ± 0.72	25.54 ± 1.57	25.66 ± 2.21	26.39 ± 1.29	**<0.001**	1 < 2, 3, 4
Global RNFL thickness, *µm*	90.6 ± 18.6	85.9 ± 10.6	73.1 ± 12.8	74.4 ± 14.2	<**0.001**	1 = 2 > 3,4
Visual field MD, *dB*	−4.72 ± 5.58	0.68 ± 0.74	−6.81 ± 5.98	−5.79 ± 6.03	<**0.001**	2 > 3,4
Visual field PSD, *dB*	3.95 ± 4.37	1.69 ± 0.56	7.00 ± 4.44	5.97 ± 4.64	<**0.001**	2 < 3, 4
Glaucoma, *n*	9 (56.3%)	7 (53.8%)	58 (90.6%)	53 (91.4%)	**0.002**	
Diabetes mellitus, *n*	0 (0%)	3 (23.1%)	11 (17.2%)	4 (6.9%)	0.079	
Systemic hypertension, *n*	2 (12.5%)	2 (15.4%)	13 (20.3%)	7 (12.1%)	0.635	
Systolic blood pressure, *mmHg*	126.9 ± 24.0	126.8 ± 15.4	128.6 ± 13.2	121.8 ± 14.3	0.106	3 > 4
Diastolic blood pressure, *mmHg*	78.5 ± 20.2	77.9 ± 7.6	78.9 ± 9.8	75.0 ± 11.2	0.319	

IOP = intraocular pressure; RNFL = retinal nerve fiber layer; MD = mean deviation; PSD = pattern standard deviation

Statistically significant values are shown in bold.

## Discussion

A small but significant increase in IOP was observed while viewing visual material on a smartphone in the present study. A regression tree was created to predict an increase in IOP associated with smartphone use, which revealed that a smaller ACD was the most significant factor for the time-dependent increase in IOP, followed by a higher VF MD, which was the most important factor in eyes with a larger ACD. Among the eyes with a larger ACD and a lower VF MD, being older was the most significant factor for an increase in IOP. This is the first study demonstrating time-dependent changes in IOP while viewing visual material on a smartphone.

The mechanism underlying the association between smartphone use and increase in IOP is unclear. One possibility is that excessive accommodation or vergence required for close working on a small screen contributed to the IOP increase^9–11^. Studies have suggested that IOP can increase with accommodation^10,11^. Yan *et al*.^10^ showed that accommodation induced a significant increase in IOP in eyes with progressive myopia, with this increase accompanied by a decrease in ACD resulting from thickening of the lens and narrowing of the anterior chamber. Liu *et al*.^11^ found that older eyes showed a significant increase in IOP with accommodation compared to younger eyes. These findings together suggest that a decrease in the ACD with accommodation is associated with a disturbed aqueous outflow resulting in an increase in IOP. Older eyes are more likely to have a thickened lens and smaller ACD, and so be more prone to increases in IOP with accommodation. This hypothesis is partly supported by the finding of a smaller ACD and older age being associated with a time-dependent increase in IOP in our study.

A regression tree analysis using a rpart tree model was used in the present study to identify the factors that best predicted the rate of IOP increase. An rpart tree model enables groups to be divided based on the factors that make different contributions to the dependent variable. The use of this model made it possible to determine how differing combinations of individual factors are associated with an IOP increase while viewing visual material on a smartphone.

The rpart tree analysis revealed that a smaller ACD was the strongest factor contributing to an IOP increase, followed by a higher VF MD, and older age. As described above, accommodation in eyes with a smaller ACD may have narrowed the anterior chamber, resulting in disturbance of the aqueous humor outflow. It also has been shown that accommodation increases the iris curvature in eyes with a narrow anterior chamber, which could induce further narrowing of the anterior chamber^18^. It is therefore possible that IOP increased significantly in such eyes even under a small amount of accommodation.

A higher VF MD was the next most significant factor for a smartphone-induced increase in IOP in eyes with a larger ACD. Eyes with a better VF may be able to focus more accurately on viewed objects, which increases the need for accommodation, and this may in turn increase the probability of an increase in IOP. It is possible that early visual fatigue induced by close working or visual neglect associated with the VF constriction in the eyes with a worse VF MD decreased the concentration required to view the visual material. Whichever the underlying cause, the relationship between the VF and accommodation remains to be determined.

When ACD was larger and the VF MD was lower, older eyes were likely to have an increased IOP when watching a movie on a smartphone. This could be linked to a previous study finding that older eyes showed an increase in IOP with accommodation^11^. It is possible that the thicker lens in older eyes influences the aqueous humor outflow for a given degree of accommodation. It is also possible that increased zonular laxity in aged eyes induced an anterior movement of lens under accommodation, which could interrupt aqueous humor outflow.

It should be noted that although younger subjects had the slowest time-dependent increase in IOP, the rate was still significantly positive. The finding indicates that watching a movie on a smartphone makes an important contribution to the IOP, and means that attention needs to be paid to the potential for an increase in IOP when users of any age view visual material on a smartphone.

This study had several limitations. Firstly, although the increase in IOP was statistically significant, its magnitude was small. Given the normal diurnal fluctuation of IOP, the increase in IOP found in the present study might not be clinically significant. However, the use of antiglaucoma eyedrops was not an exclusion criterion in the current study, and so their presence might have affected the IOP responses during the intervention. Moreover, it should be kept in mind that even a small change in IOP could greatly affect the disease prognosis specifically in patients with advanced glaucoma. Secondly, the structure of the anterior chamber could not be examined during the fixation task. A recent study demonstrated a transient IOP increase with a change in the anterior segment biometry during accommodative stimuli^10^. Imaging the anterior segment structure during smartphone use could clarify the relationships between accommodation, changes in the anterior chamber angle, and IOP increases. Thirdly, the visual angle between individual eyes and smartphone screen, or sitting posture of individual subjects such as body and head position also could have influenced IOP^19–21^, which were not controlled in our study. However, we believe that neither could fully explain the time-dependent manner of IOP elevation observed in the present study. Fourthly, experimental procedure was performed during daytime, and so our results may not be applicable for nighttime smartphone usage. Circadian change of IOP is a well-documented phenomenon^14–16^, thus it would also be interesting to know how day- and nighttime smartphone usage differs in terms of their influence on IOP. Fifthly, subjects used their habitual refractive correction during the examination to best reproduce their habitual circumstances. However, it is possible that subjects did not achieve their best corrected near vision, which might have affected the degree of accommodation. Lastly, the participants in the present cohort were ethnically homogeneous Koreans, and so our findings might not be generalizable in other ethnic groups.

In conclusion, a small but significant increase in IOP was observed while viewing visual material on a smartphone. A shallow anterior chamber and high VF sensitivity were strongly predictive of the time-dependent increase in IOP during smartphone use. These findings may help to modify the lifestyle factors significant to smartphone use specifically in patients with glaucomatous damage at risk of progression due to the potential importance of small changes in IOP.

## Methods

The participants in this study comprised consecutive patients with primary open-angle glaucoma (POAG) and healthy subjects who visited the Glaucoma Clinic of Seoul National University Bundang Hospital from August 2016 to November 2017. All of the included subjects provided written informed consent to participate. This study was approved by the Institutional Review Board of Seoul National University Bundang Hospital and followed the tenets of the Declaration of Helsinki.

### Baseline ophthalmic examination

Subjects underwent a comprehensive ophthalmic examination, including visual acuity assessment, Goldmann applanation tonometry, refraction tests, slit-lamp biomicroscopy, gonioscopy, dilated stereoscopic examination of the optic disc, disc photography (EOS D60 digital camera, Canon, Utsunomiyashi, Tochigiken, Japan), spectral-domain optical coherence tomography (SD-OCT) circumpapillary retinal nerve fiber layer (RNFL) scanning (Spectralis, Heidelberg Engineering, Heidelberg, Germany), standard automated perimetry (Humphrey Field Analyzer II 750 and 24-2 Swedish interactive threshold algorithm, Carl Zeiss Meditec, Dublin, CA, USA), and measurements of corneal curvature (KR-1800, Topcon, Tokyo, Japan), central corneal thickness, anterior chamber depth (ACD; Orbscan II, Bausch & Lomb Surgical, Rochester, NY, USA), and axial length (IOL Master version 5, Carl Zeiss Meditec)^22^.

The systolic and diastolic blood pressures (BPs) were measured using a digital automatic BP monitor (Omron HEM-770A, Omron Matsusaka, Matsusaka, Japan)^22^. The mean arterial pressure (MAP) was calculated using as diastolic BP + 1/3(systolic BP – diastolic BP), and the mean ocular perfusion pressure was calculated as 2/3(MAP – IOP)^23^.

Subjects were excluded when they had a history of intraocular surgery including cataract extraction, an intraocular disease (e.g., diabetic retinopathy or retinal vein occlusion), a neurologic disease (e.g., pituitary tumor) that could cause visual field loss, or a visual acuity worse than 20/40.

POAG was defined as the presence of glaucomatous optic nerve damage (e.g., the presence of focal thinning, notching, and an RNFL defect), an associated glaucomatous visual field (VF) defect, and an open angle, as revealed by gonioscopy. A glaucomatous VF was defined as (1) values outside the normal limits on the glaucoma hemifield test; (2) three abnormal points, with a probability of being normal of *P* < 0.05, and one point with a pattern deviation of *P* < 0.01; or (3) a pattern standard deviation of P < 0.05. These VF defects needed to be confirmed in two consecutive reliable tests (fixation loss rate, ≤20%; false-positive and false-negative error rates, ≤25%)^24^.

Healthy subjects had IOP ≤ 21 mmHg with no history of an increased IOP, the absence of a glaucomatous disc, a circumpapillary RNFL thickness within the normal range (as measured by SD-OCT), and a normal VF. The absence of a glaucomatous disc was defined as an intact neuroretinal rim without peripapillary hemorrhages, notches, or localized pallor. The normal range for the RNFL thickness was defined as circumpapillary RNFL thicknesses within the 95th percentile of the normative database, while the VF was considered normal when there was no glaucomatous VF defect or neurologic defect.

### Equipment

The experimental fixation task applied in this study required 30 minutes of continuous smartphone viewing. A Galaxy S5 smartphone (Samsung Electronics, Seoul, Korea) was used to display visual material. This device has a liquid crystal display touchscreen with a display size of 5.1 inches and a resolution of 1080 pixels × 1920 pixels.

The displayed visual material was a commercially available movie animation. The luminance of the screen was adjusted to 50% of the maximum backlight luminance.

### Experimental procedure

Subjects were required to perform a fixation task consisting of watching a movie on a smartphone screen for 30 minutes continuously at a viewing distance of 30 cm. Where applicable, subjects wore their habitual refractive correction while viewing the material.

IOP was measured before starting the fixation task, and then repeated at 5, 10, and 30 minutes after starting the task. All subjects had to close their eye for at least five minutes before the baseline IOP measurement. IOP was measured using an iCare rebound tonometer (iCare Company, Vantaa, Finland) by a single examiner who was masked to the clinical information of the participants.

### Statistical analysis

A linear mixed-effects model for repeated-measures data was used to assess the time-dependent change in IOP during smartphone use^25^. The rpart package (version 3.1-3) in the R programming environment (R version 2.2; R-Project, available at http://cran.r-project.org) was used to construct regression trees in order to identify the factors that best described any IOP change during the intervention^17^. The subgroups classified from the rpart tree analysis were compared using analysis of variance with the Bonferroni post-hoc test.

Statistical analyses other than the rpart tree analysis were performed using the Statistical Package for the Social Sciences software (version 22.0, SPSS, Chicago, IL, USA). Probability values of *P* < 0.05 were considered indicative of statistical significance. Except where stated otherwise, data are presented as mean ± SD values.

## Supplementary information

Supplementary Dataset 1

## Data Availability

Data supporting the findings of the current study are available in Supplementary File.

## References

[1] Wikipedia. List of countries by smartphone penetration, https://en.wikipedia.org/wiki/List_of_countries_by_smartphone_penetration.

[2] Statista. Statistics on smartphones, http://bit.ly/1l4ehuw.

[3] Antona B (2018). Symptoms associated with reading from a smartphone in conditions of light and dark. Applied ergonomics.

[4] Lee S, Kang H, Shin G (2015). Head flexion angle while using a smartphone. Ergonomics.

[5] Kim J (2016). Association between Exposure to Smartphones and Ocular Health in Adolescents. Ophthalmic Epidemiol.

[6] Kucer N (2008). Some ocular symptoms experienced by users of mobile phones. Electromagn Biol Med.

[7] Moon JH, Lee MY, Moon NJ (2014). Association between video display terminal use and dry eye disease in school children. J Pediatr Ophthalmol Strabismus.

[8] Balik HH, Turgut-Balik D, Balikci K, Ozcan IC (2005). Some ocular symptoms and sensations experienced by long term users of mobile phones. Pathol Biol (Paris).

[9] Bababekova Y, Rosenfield M, Hue JE, Huang RR (2011). Font size and viewing distance of handheld smart phones. Optom Vis Sci.

[10] Yan L, Huibin L, Xuemin L (2014). Accommodation-induced intraocular pressure changes in progressing myopes and emmetropes. Eye (Lond).

[11] Liu Y (2015). Intraocular Pressure Changes during Accommodation in Progressing Myopes, Stable Myopes and Emmetropes. PLoS One.

[12] Osborne NN, Lascaratos G, Bron AJ, Chidlow G, Wood JP (2006). A hypothesis to suggest that light is a risk factor in glaucoma and the mitochondrial optic neuropathies. Br J Ophthalmol.

[13] Liu JH, Shieh BE, Alston CS (1994). Short-wavelength light reduces circadian elevation of intraocular pressure in rabbits. Neurosci Lett.

[14] Maeda A (2006). Circadian intraocular pressure rhythm is generated by clock genes. Invest Ophthalmol Vis Sci.

[15] Rowland JM, Potter DE, Reiter RJ (1981). Circadian rhythm in intraocular pressure: a rabbit model. Curr Eye Res.

[16] Liu JH (1998). Nocturnal elevation of intraocular pressure in young adults. Invest Ophthalmol Vis Sci.

[17] Therneau, T. M. & Atkinson, E. J. Technical Report 61, http://www.mayo.edu/hsr/techrpt/61.pdf (1997).

[18] Dorairaj S (2008). Accommodation-induced changes in iris curvature. Exp Eye Res.

[19] Prata TS, De Moraes CG, Kanadani FN, Ritch R, Paranhos A (2010). Posture-induced intraocular pressure changes: considerations regarding body position in glaucoma patients. Surv Ophthalmol.

[20] Malihi M, Sit AJ (2012). Effect of head and body position on intraocular pressure. Ophthalmology.

[21] Ha A, Kim YK, Park YJ, Jeoung JW, Park KH (2018). Intraocular pressure change during reading or writing on smartphone. PLoS One.

[22] Lee EJ (2019). Elucidation of the Strongest Factors Influencing Rapid Retinal Nerve Fiber Layer Thinning in Glaucoma. Invest Ophthalmol Vis Sci.

[23] Kiel JW, van Heuven WA (1995). Ocular perfusion pressure and choroidal blood flow in the rabbit. Invest Ophthalmol Vis Sci.

[24] Caprioli J (1991). Automated perimetry in glaucoma. Am J Ophthalmol.

[25] Lindstrom, M. J. & Bates, D. M. Nonlinear mixed effects models for repeated measures data. *Biometrics*, 673–687 (1990). 2242409

